# Defect of branched-chain amino acid metabolism promotes the development of Alzheimer’s disease by targeting the mTOR signaling

**DOI:** 10.1042/BSR20180127

**Published:** 2018-07-03

**Authors:** Huajie Li, Dan Ye, Wei Xie, Fei Hua, Yilin Yang, Jian Wu, Aifang Gu, Yi Ren, Keshi Mao

**Affiliations:** 1 Department of Neurology, the First People’s Hospital of Chang Zhou, Jiang Su, China; 2 The Third Affiliated Hospital of Soochow University; 3 Department of Endocrinology, the First People’s Hospital of Chang Zhou, Jiang Su, China; 4 Department of Neurosurgery, the First People’s Hospital of Chang Zhou, Jiang Su, China

**Keywords:** AD, BCAA, BCAT1, Diabetes, mTOR, Tau

## Abstract

Diabetes is a risk factor for Alzheimer’s disease (AD) in humans. Branched-chain amino acids (BCAAs, namely valine, leucine, and isoleucine) metabolic defect is observed in human diabetes, which is associated with insulin resistance. But whether BCAAs connect diabetes and AD remains unknown. Here, we show that BCAA metabolic defect may be one of the drivers of AD. BCAA levels were increased in the blood in human patients and mice with diabetes or AD. BCAA-enriched diet promoted the development of AD in mice as evidenced by the behavior and pathological analysis. Branched-chain amino acid transaminase 1 and 2 (BCAT1 and BCAT2) are the two enzymes for the first step metabolism of BCAAs by catalyzing BCAAs to generate branched-chain ketoacids. The expression of *Bcat1* but not *Bcat2* was significantly down-regulated in the brain tissues of diabetic, aged, and AD mice. Leucine up-regulated the phosphorylation of Tau but not affected the accumulation of amyloid β in the brain tissues or isolated neurons. In addition, knockdown of the expression of *Bcat1*, which would result in the accumulation of BCAAs, led to the same phenotype as BCAAs supplement in neurons. Interestingly, leucine supplement or *Bcat1* knockdown promoted the activation of the mTOR signaling in the brains of AD mice or neurons. Subsequently, mTOR was critically involved in leucine and *Bcat1* knockdown-mediated phosphorylation of Tau. Taken together, our findings demonstrated that diabetes-related BCAA accumulation in the brain tissues led to the phosphorylation of Tau and, subsequently, the development of diabetes-related AD.

## Introduction

Alzheimer’s disease (AD) is one of the most prevalent causes of dementia. Amyloid β42 and phosphorylated Tau proteins are established as core cerebrospinal biomarkers for AD [1]. However, treatment strategies targeted at reducing amyloids have failed to reverse cognitive symptoms. Cognitive decline is the result of complex pathophysiology and targeting amyloid-alone may not be sufficient to treat AD [2,3]. Instead, a broad outlook on neural-circuit-damaging processes may yield insights into new therapeutic strategies for curing memory loss in the disease.

AD is fundamentally a metabolic disease with molecular and biochemical features that correspond with diabetes and other peripheral insulin resistance disorders [4]. Advanced glycation end products generated by chronic hyperglycemia and their receptor for advanced glycation end products (RAGE) provide critical links between diabetes and AD [5]. Continued elucidation of the links between metabolic disorders and neural dysfunction promises to foster the development of effective therapeutic strategies [6]. However, diabetes is connected to AD clinically, and experimental molecular and biochemical links are limited. One of the evidence for connecting diabetes and AD is the signaling factor mammalian target of rapamycin (mTOR). mTOR was already well known as a regulator of growth and protein translation, it is now clear that mTOR functions as a central coordinator of organismal metabolism in response to both environmental and hormonal signals, and it involved in the development of diabetes [7]. Interestingly, vascular mTOR-dependent mechanisms linking the control of aging to Alzheimer’s disease [8]. mTOR is core regulator and effector of insulin signaling. Our prior work indicates that insulin degrading enzyme (IDE) contributes to the pathology in a mixed model of type 2 diabetes and AD [9], and that adenosine 3,5′-cyclic monophosphate/protein kinase A (cAMP/PKA) signaling pathway contributes to neuronal apoptosis via regulating IDE expression in a mixed model of type 2 diabetes and AD [10]. In addition, we showed that glucagon-like peptide 1 (GLP-1) receptor regulates cell growth through regulating IDE expression level in Aβ1-42-treated PC12 cells [11]. However, the mechanism connecting diabetes and AD is still not fully understood.

Branched-chain amino acids (BCAAs, namely leucine, isoleucine, and valine) are amino acids having aliphatic side chains with a branch (a central carbon atom bound to three or more carbon atoms). The first step in the metabolism of BCAAs in most peripheral tissues, except the liver, is catalyzed by the cytosol or mitochondrial isoform of branched-chain amino acid transaminase (BCAT1 and BCAT2). After BCAT, the next step in the BCAA metabolic pathway is rate controlling and the first irreversible step in BCAA metabolism [12]. BCAAs are recently reported to be associated with human diseases including cancer [13,14], diabetes [15,16], and cardiovascular diseases [17,18]. The use of high-throughput metabolomics allowed us to uncover the associations between the concentrations of BCAAs in blood and predisposition to diabetes and insulin resistance in human [15,19]. However, the functions of BCAAs and their metabolic enzymes in human neurons and AD remain unknown.

Here, in the present work, we showed that BCAAs are tightly associated with diabetes and AD. The plasma levels of BCAAs were significantly increased in human diabetic and AD patients as well as old individuals. Supplement of BCAAs in diet promoted the cognitive deficits in 3xTg-AD mice. BCAA accumulation may be due to the down-regulation of *Bcat1* in the brains. *Bcat1* down-regulation led to leucine accumulation, which promoted the phosphorylation of Tau protein in an mTOR-dependent manner.

## Materials and methods

### Patients

Diabetic and AD patients, as well as young and old healthy donors, were recruited at the First People’s Hospital of Changzhou. A written form of consent was obtained from all patients and donors. Blood samples were collected from the patients and donors and stored at −80°C before use. The study was approved by the clinical research ethics committee of the First People’s Hospital of Changzhou.

### Mice

The triple transgenic Alzheimer disease (3xTg-AD) mice (Stock number 34830) were purchased from Jackson Lab. Aged (24-month-old) and *db/db* diabetic mice were purchased from Charles River Laboratories. Animals were given unrestricted access to a standard diet (4.3 kcal % fat, 18.8 kcal % protein, and 76.9 kcal % carbohydrate) and tap water. For wild-type and 3xTg-AD, mice were randomly assigned to control and BCAA-supplemented groups (1.5 mg/g body weight/day) in drinking water. BCAA supplementation was performed from 3-month-old for 3 months. For functional study, the behavior and memory defects of the animals were analyzed at 6 months. For Western blot and amyloid content analysis, the mice were killed and the brain tissues were subjected to protein extraction and Western blot or amyloid content measurement. The animal experiments were approved by the animal research ethics committee of the First People’s Hospital of Changzhou.

### Neuron isolation and culture

Neurons were isolated from adult male wild-type or 3xTg AD mice using a Pierce Primary Neuron Isolation Kit (Thermo Fisher, 88280).

### Measurement of BCAAs in the serum

The levels of BCAAs in the serum were measured as described previously [14], using liquid chromatography-tandem mass spectrometry (LC-MS/MS) at the Core Laboratory of Soochow University. Targeted LC-MS/MS was performed with standard valine, isoleucine, and leucine samples.

### Western blot

Total proteins were extracted from brain tissues and neurons with RIPA buffer (Beyotime, P0013) supplied with proteinase inhibitor cocktail (Roche, 04693124001) and phosphatase inhibitor cocktail (Sigma, P5726). Total protein (30 μg) was subjected to SDS-PAGE for protein separation. The proteins were transferred to PVDF membranes and blocked with 5% fat-free milk in TBST buffer, and then the membranes were incubated with individual primary antibodies overnight. Then the membranes were washed and incubated with horseradish peroxidase (HRP)-conjugated secondary antibodies (Zhongshanjinqiao) for 2 h. Finally, the secondary antibodies were detected by SuperSignal™ Chemiluminescent HRP Substrates (Thermo Fisher, 32106). The following primary antibodies were used in the present study: anti-GAPDH antibody (Santa Cruz, sc-47724), anti-BCAT1 antibody (Novus Biological, NBP2-01826), anti-Tau antibody (Abcam, ab64193), anti-p-Tau antibody (Abcam, ab109390), anti-mTOR antibody (Cell Signaling Technology, 2983), anti-p-mTOR antibody (Cell Signaling Technology, 5536), anti-S6K1 antibody (Cell Signaling Technology, 9202), and anti-p-S6K1 antibody (Cell Signaling Technology, 9206).

### Quantitative real-time PCR

Total RNAs were isolated from brain tissues and neurons with TRIzol reagent (Thermo Fisher, 15596026). Then 2 μg of total RNA was subjected to cDNA synthesis with the First Strand cDNA Synthesis Kit (Thermo Fisher, K1612). Next, the relative mRNA levels of targeted genes were analyzed by quantitative real-time PCR (qPCR) with iQ™ SYBR® Green Supermix (Bio-Rad, 1708880). The primers used for qPCR were as follows:

*Bcat1* forward: 5′-GAAGTGGCGGAGACTTTTAGG-3′
*Bcat1* reverse: 5′-TGGTCAGTAAACGTAGCTCCA-3′
*Bcat2* forward: 5′-AAAGCATACAAAGGTGGAGACC-3′
*Bcat2* reverse: 5′-CGTAGAGGCTCGTTCCGTTG-3′
*Gapdh* forward: 5′-AATGGATTTGGACGCATTGGT-3′
*Gapdh* reverse: 5′-TTTGCACTGGTACGTGTTGAT-3′

### Lentivirus packaging

To knockdown the expression of mouse *Bcat1*, short-hairpin RNAs (shRNAs) targeting *Bcat1* mRNA (sh*Bcat1*) were purchased from Invitrogen and expressed with lentivirus. The shRNA sequences targeting *Bcat1* are as follows: sh*Bcat1*: 5′-GGGCCAAAGATCTCATCATCA-3′. The lentivirus was prepared as described previously [20].

### Amyloid content measurement

The accumulation of amyloid in the neurons was analyzed with the β-Amyloid (1-42) ELISA Kit (Invitrogen, 99-0064).

### Y-maze alternation task

The total number of arm entries was recorded, as it was the number of entries representing alternation behavior (i.e. sequential entry into all three arms). All four paws of the mouse had to enter an arm for it to count as an arm entry. The percentage spontaneous alternation = (number of alternations)/(total arm entries − 2).

### Morris water maze

The reference memory version of the MWM task was performed as described previously [1]. Briefly, animals were trained to swim in a 1.4 m diameter pool to find a submerged platform located 1 cm below the surface of water (24°C), rendered opaque by the addition of nontoxic white paint. Animals were pseudorandomly started from a different position at each trial and used distal visual spatial cues to find the hidden escape platform that remained in the center of the same quadrant throughout all training days. Training measures included escape latency to reach the platform and swim speed. To assess visual deficits and motivation to escape from water, the probe test was followed by a cued task during which the platform was visible. The visible platform was moved to different locations between each trial. After each trial, animals were immediately placed under a warming lamp to dry to prevent hypothermia.

### Statistical analysis

The values are presented as the mean ± SEM of at least three independent repeats. Two-tail unpaired or paired Student’s *t*-test was applied to analyze the differences between two groups. Differences among groups were determined by one-way or two-way analysis of variance (ANOVA) with/without repeated measures, followed by the Bonferroni *post-hoc* test. *P* value of less than 0.05 was considered significant. All normalized data were normalized to the control group, which was considered as 1 or 100%. The statistical analyses were performed using GraphPad Prism 7.0.

## Results

### BCAAs accumulate in the serum of diabetic, aged, and AD patients and mice

**Figure 1:**
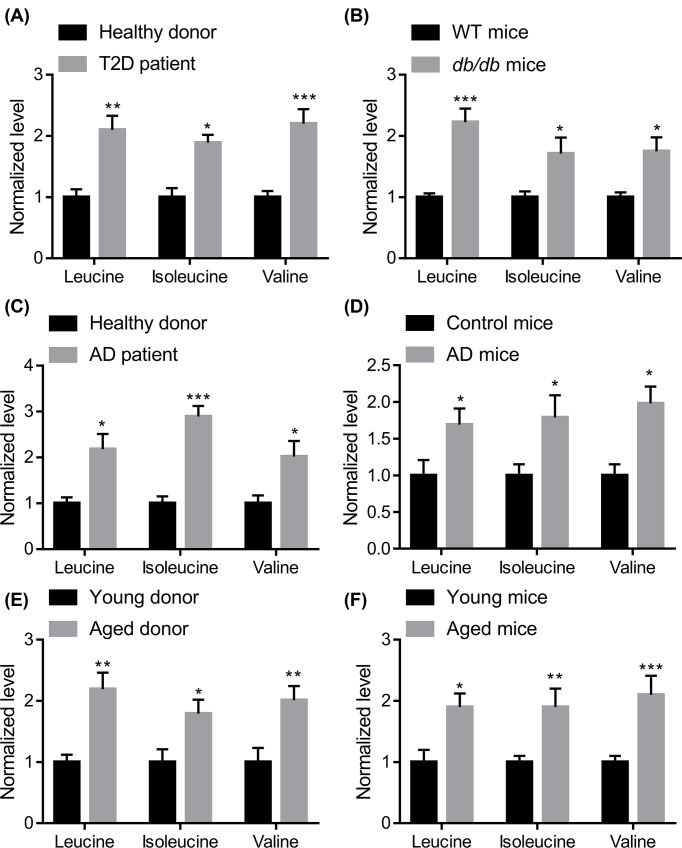
BCAAs accumulate in the blood of human and mouse diabetes and Alzheimer’s disease (**A**) BCAA levels were increased in the serum of diabetic patients (*n*=10; 6 males and 4 females; mean age = 69.1 years; 4 with and 6 without Alzheimer’s disease) compared with age-matched healthy donors (*n*=6; 3 males and 3 females; mean age = 67.7 years; without AD); T2D, type 2 diabetes. (**B**) BCAA levels were increased in the serum of *db/db* diabetic mice (*leptin receptor* mutant mice) compared with age-matched littermates (*n*=5). All the mice were males and 4 months old. (**C**) BCAA levels were increased in the serum of patients with AD (*n*=8; 5 males and 3 females; mean age = 71.3 years; 4 with and 4 without diabetes) compared with age-matched healthy donors (*n*=6; 3 males and 3 females; mean age = 69.7 years; without diabetes). (**D**) BCAA levels were increased in the serum of triple *APP_swe_, PS1_M146V_*, and *Tau_P301L_* transgenic (3xTg) AD mice compared with age-matched littermates (*n*=5). All the mice were males and 6 months old. (**E**) BCAA levels were increased in the serum of aged donors (*n*=8; 5 males and 3 females; mean age = 76.3 years; 2 with diabetes and AD, 1 with diabetes and 1 with AD and 4 without diabetes or AD) compared with young donors (*n*=6; 4 males and 2 females; mean age = 34.5 years; without diabetes or AD). (**F**) BCAA levels were increased in the serum of aged (24-month-old) male mice compared with young (4-month-old) male mice (*n*=5); *P<0.05, **P<0.01, and ***P<0.001 by unpaired Student’s *t*-test.

BCAAs are associated with the development of diabetes [15]. We aimed to investigate whether BCAAs are a factor connecting diabetes and AD. The plasma levels of BCAAs in healthy donors and diabetic patients were determined. The results showed that BCAA levels were increased in the serum of patients with type 2 diabetes (*n*=10; 6 males and 4 females; mean age = 69.1 years; 4 with and six without Alzheimer’s disease) compared with aged-matched healthy donors (*n*=6; 3 males and 3 females; mean age = 67.7 years; without AD) (Figure 1A). We also tested the plasma BCAA levels in wild-type mice and *db/db* mutant diabetic mice (4-month-old) and the results also showed that BCAA levels were increased in diabetic mice (Figure 1B). Next, we analyzed the plasma levels of BCAAs in AD patients (*n*=8; 5 males and 3 females; mean age = 71.3 years; 4 with and 4 without diabetes) compared with age-matched healthy donors (*n*=6; 3 males and 3 females; mean age = 69.7 years; without diabetes). The results revealed that the plasma BCAA levels were up-regulated in AD patients (Figure 1C). The up-regulation of BCAA levels in the plasma was also observed in the triple APP_swe_, PS1_M146V_, and Tau_P301L_ transgenic (3xTg) AD model compared with nontransgenic mice (Figure 1D). Aging is one of the core risk factors for diabetes and AD. Plasma levels of BCAAs were significantly up-regulated in aged donors (*n*=8; 5 males and 3 females; mean age = 76.3 years; 2 with diabetes and AD, 1 with diabetes and 1 with AD, and 4 without diabetes or AD) and mice (24-month-old) (Figure 1E,F). In addition, we also tested the level of lysine in the serum of T2D, AD, and aged donors, not significant change in lysine serum level was observed (Supplementary Figure S1). Taken together, the up-regulation of BCAAs in the serum may connect diabetes and AD in human and mice.

### BCAA diet increases cognitive deficits in 3xTg-AD mice

**Figure 2:**
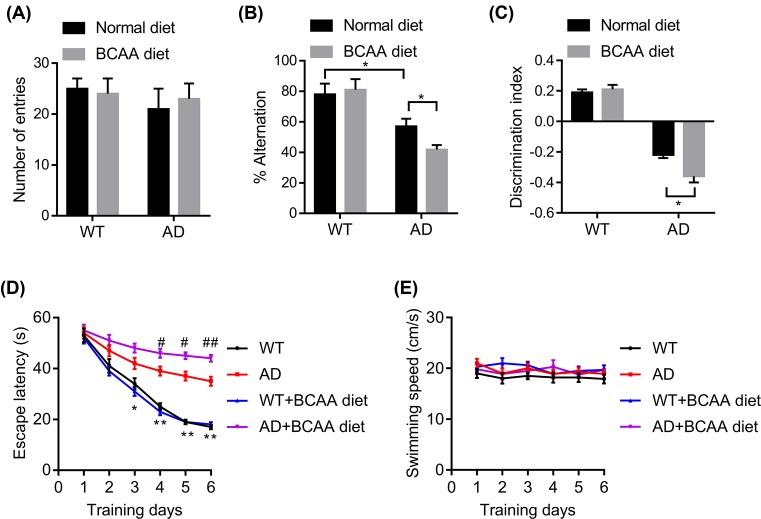
BCAAs diet promotes the development of AD in mice WT and 3xTg-AD mice (male, 6-month-old) were treated with normal or BCAA diet and tested in the Y-maze, novel object recognition (NOR), and Morris water maze (MWM) tasks. (**A** and **B**) Y-maze, a number of arm entries and percentage spontaneous alternations were calculated (*n*=8, *p<0.05 by two-way ANOVA followed by Bonferroni *post-hoc* test). (**C**) NOR, the discrimination index of each group, was calculated (*n*=8, *p<0.05 by two-way ANOVA followed by Bonferroni *post-hoc* test). (**D**) MWM, the 3xTg-AD mice, fed with BCAA diet showed longer escape latency before finding the hidden platform (3 trials/day; 60 s; 30 m intertrial interval) when compared with AD mice fed with normal diet (*n*=8, *P<0.05, **PP<0.01 indicates WT compared with AD; ^#^*P*<0.05, ^##^*P*<0.01 indicates AD BCAA diet compared with AD normal diet by repeated two-way ANOVA followed by Bonferroni *post-hoc* test). (**E**) Swim speed at each training day was not significantly different between groups (*n*=8).

We next tested whether BCAAs supplement in the diet would affect the cognitive deficits in an AD mouse model. The 3xTg AD mice and nontransgenic wild-type mice were fed with a normal diet or BCAA diet for 3 months. We used the Y-maze to evaluate spatial working memory function in 3xTg-AD mice. Spontaneous alternations and total arm entries were calculated. There was no significant change in the arm entries in BCAAs-fed AD mice, indicating that the BCAAs did not affect general motor activity (Figure 2A). However, the spontaneous alternations indicated that the AD mice fed with BCAA diet made more incorrect choices compared with AD mice fed with normal diet (Figure 2B). Next, we also investigated whether BCAA diet reduced performance in the novel object recognition (NOR) task. Wild-type and AD mice had no significant differences in baseline locomotor activity as measured during the habituation phase. Interestingly, the discrimination index in the AD–BCAA group was significantly lower than those of the AD fed with normal diet (Figure 2C). Then we analyzed the effects of BCAA diet in the reference memory version of the Morris water maze (MWM). BCAA diet promoted the memory deficits in AD mice on day 4, 5, and 6 of the acquisition phase (Figure 2D). The longer escape latency of AD mice fed with BCAA diet was not attributed to slower swimming speed, as no significant difference was found between groups (Figure 2E). Collectively, our data revealed that BCAA diet increased cognitive deficits in AD mice, which indicates that BCAAs may connect diabetes and AD.

### BCAT1 down-regulation activates the phosphorylation of Tau

**Figure 3:**
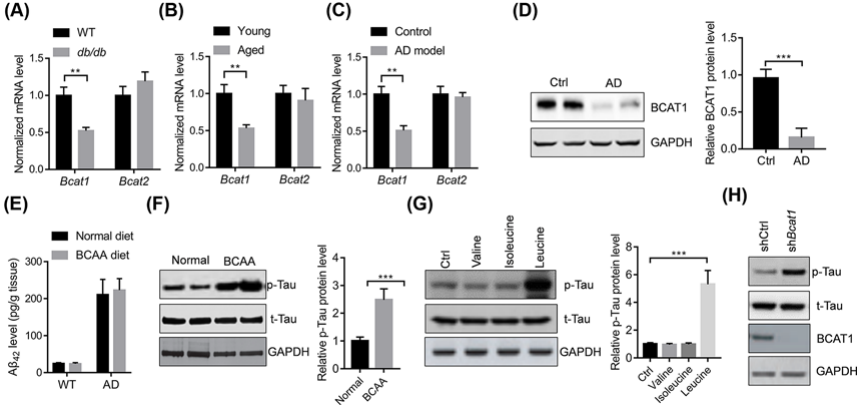
BCAAs promote the phosphorylation of Tau (**A–C**) *Bcat1* mRNA level is decreased in the brain tissues of diabetic, aged, or 3xTg AD mice (*n*=5). **P<0.01 by unpaired Student’s *t*-test. (**D**) Representative Western blot and quantitative results showing BCAT1 protein level are decreased in the brain tissues of AD mice (*n*=4). ***P<0.001 by unpaired Student’s *t*-test. (**E**) BCAA diet does not affect the content of amyloid β42 (Aβ42) in the brain tissues of AD mice (*n*=5). (**F**) Representative Western blot and quantitative results showing BCAA diet increase the level of phosphorylated Tau protein in the brain tissues of AD mice (*n*=4). ***p<0.001 by unpaired Student’s *t*-test. (**G**) Relative Western blot and quantitative results showing leucine increase the level of phosphorylated Tau protein in the neurons isolated from mice. The neurons were isolated from the 3xTg mice and treated with leucine (1 mM), isoleucine (1 mM), or valine (1 mM) for 24 h. The experiments were repeated for three times. ***P<0.001 by one-way ANOVA followed by Bonferroni *post-hoc* test. (**H**) *Bcat1* knockdown increased the level of phosphorylated Tau protein in the neurons isolated from mice. The neurons were isolated from the 3xTg mice and infected with lentivirus carrying indicated shRNAs for 48 h.

BCAA accumulation may be affected by the branched-chain amino acid transaminase 1 (BCAT1) and BCAT2. Therefore, we analyzed the expression of *Bcat1* and *Bcat2* in the brain tissues of diabetic, aged, and AD mice. The results showed that the mRNA and protein levels of *Bcat1* but not *Bcat2* were significantly down-regulated in the brain tissues of diabetic, aged, and AD mice compared with their control mice (Figure 3A–D).

As one of the hallmarks of AD, extracellular accumulation of β-amyloid peptides has been a common target. We, therefore, analyzed whether *Bcat1* down-regulation and subsequent accumulation of BCAAs in the brain tissues affect the content of β-amyloid peptides in the AD mice. The results showed that BCAA diet did not affect the content of β-amyloid peptides in the brain of AD mice (Figure 3E). Another hallmark of the AD is hyperphosphorylation and accumulation of the Tau protein, a microtubule-associated protein known to play a role in axonal growth and plasticity [1]. We found that BCAA diet significantly increased the phosphorylated level of Tau in the brain tissues of the AD mice (Figure 3F). Next, we also analyzed which amino acid affected the accumulation of phosphorylated Tau protein in isolated neurons. The results showed that leucine but not isoleucine or valine up-regulated the level of phosphorylated Tau protein in neurons isolated from 3xTg mice (Figure 3G). We also knocked down the expression of *Bcat1* with lentivirus in isolated neurons of AD mice and found that *Bcat1* knockdown up-regulated the levels of phosphorylated levels of Tau in neurons (Figure 3H).

### BCAA activates p-Tau in an mTOR-dependent manner

**Figure 4:**
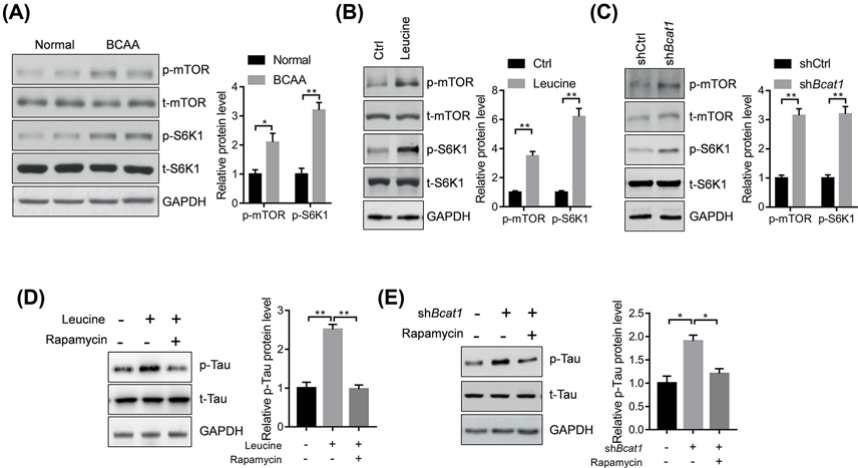
BCAAs promote the p-Tau in an mTOR-dependent manner (**A**) Representative Western blot and quantitative results showing BCAA diet activated mTOR signaling in the brain tissues of 3xTg AD mice (*n*=4, *P<0.05, **P<0.01 by unpaired Student’s *t*-test). (**B**) Representative Western blot and quantitative results showing leucine activated mTOR signaling in the neurons isolated from mice. The neurons were isolated from the 3xTg AD mice and treated with leucine (1 mM) for 24 h. The experiments were repeated for three times. **P<0.01 by unpaired Student’s *t*-test. (**C**) Representative Western blot and quantitative results showing *Bcat1* knockdown activated mTOR signaling in the neurons isolated from mice. The neurons were isolated from the 3xTg AD mice and infected with lentivirus carrying indicated shRNAs for 48 h. The experiments were repeated for three times. **P<0.01 by unpaired Student’s *t*-test. **(D**) Representative Western blot and quantitative results showing inhibition of mTOR with rapamycin repress leucine-mediated increase in phosphorylated Tau protein in neurons isolated from mice. The neurons were isolated from the 3xTg AD mice and treated with leucine (1 mM) and rapamycin (10 nM) for 24 h. The experiments were repeated for three times. **P<0.01 by one-way ANOVA followed by Bonferroni *post-hoc* test. (**E**) Representative Western blot and quantitative results showing inhibition of mTOR with rapamycin repress *Bcat1* knockdown-mediated increase in phosphorylated Tau protein in neurons isolated from mice. The neurons were isolated from the 3xTg AD mice and infected with lentivirus carrying indicated shRNAs in the presence of rapamycin (10 nM) for 48 h. The experiments were repeated for three times. **P<0.01 by one-way ANOVA followed by Bonferroni *post-hoc* test.

Finally, we analyzed the potential mechanism underlying leucine and BCAT1 effects on the phosphorylated level of Tau in brain tissues and neurons of AD mice. mTOR was reported to phosphorylate Tau [21]. We tested whether BCAA diet increased the activation of mTOR signaling in the brain tissues of AD mice and found that BCAA diet increased the phosphorylation levels of mTOR and its downstream target S6K1 (Figure 4A). Leucine treatment or *Bcat1* knockdown also promoted the activation of the mTOR–S6K1 signaling (Figure 4B,C). Finally, we analyzed whether the mTOR signaling was critically involved in the effects of leucine and *Bcat1* on phosphorylation of Tau. Therefore, we treated the neurons isolated from the AD mice with mTOR inhibitor rapamycin. The results showed that rapamycin treatment blocked the leucine- or *Bcat1* knockdown-mediated accumulation of phosphorylated Tau in neurons (Figure 4D,E). Therefore, BCAAs activated p-Tau in an mTOR-dependent manner.

## Discussion

Metabolic diseases are often present for years before becoming clinically apparent. BCAAs have highly significant association with future diabetes [15] and are associated with improvement in insulin resistance with weight loss [22]. Elevations in the concentrations of circulating BCAAs are significantly associated with obesity in children, adolescents, and young adults, which may independently predict future insulin resistance [23,24]. BCAA metabolism is also associated with neurological disorders. Mutations in BCKD-kinase lead to a potentially treatable form of autism with epilepsy, which is associated with the defect in BCAA metabolism [25]. In addition, impaired BCAA transport at the blood–brain barrier is another cause of autism spectrum disorder [26].

Since diabetes is tightly related to the development of AD and BCAA, metabolism is associated with diabetes and neurological disorders as we studied the potential roles of BCAA in the AD. We first analyzed the plasma levels of BCAA in diabetic, aged, or AD individuals in human and mice. Results showed that plasma levels of BCAAs were significantly up-regulated in the diabetic, aged, or AD individuals. High levels of plasma BCAAs are reported to be associated with insulin resistance [12], which is considered as a fundamental linker between diabetes and AD [27]. These findings reveal that BCAAs may connect diabetes, aging, and AD.

To explore the effects of high BCAA levels on the cognitive deficits of AD mice, we fed the 3xTg AD mice with BCAA diet and analyzed the behavior performance or memory capacity of these mice. Compared with 3xTg AD mice fed a normal diet, the BCAAs-fed AD mice showed more serious memory defects as evidenced by decreased alternation, negative discrimination index, and longer escape latency in MWM test. However, BCAA supplement alone did not change the general behaviors of wild-type mice, indicating that high BCAAs alone may be not a driver of the AD. In addition, we only found that BCAAs changed in patients with diabetes and AD. We did not obtain any evidence that BCAAs were the core connecting factor between diabetes and AD. Further, studies are needed to elucidate whether BCAAs were critically involved in the pathological process of the diabetic AD.

In contrast with many other factors that may regulate the risk of the AD, BCAAs are particularly susceptible to dietary modulation. Protein-restricted, high-carbohydrate diets improve metabolic health in rodents. Decreasing dietary BCAAs has been shown to improve metabolic health in rodents. Feeding mice a diet specifically reduced in BCAAs is sufficient to improve glucose tolerance and body composition equivalently to a PR diet via metabolically distinct pathways [28]. BCAA restriction in Zucker-fatty rats improves muscle insulin sensitivity by enhancing the efficiency of fatty acid oxidation and acyl-glycine export [29]. A reduced BCAA diet promotes rapid fat mass loss without calorie restriction in obese mice. Selective reduction in dietary BCAAs also restores glucose tolerance and insulin sensitivity to obese mice, even as they continue to consume a high-fat, high-sugar diet [30]. In addition, plasma isoleucine levels have been genetically linked with the risk of AD [31]. Our data also demonstrated the plasma BCAAs were increased in AD and BCAAs promoted the development of AD. Therefore, protein-restricted diet or BCAAs-restricted diet may serve as a potential approach for AD treatment.

BCAA supplement showed varying results in different conditions. Long-term supplement of BCAAs in the diet was reported to promote survival and support cardiac and skeletal muscle mitochondrial biogenesis in middle-aged mice [32]. However, others reported that BCAA supplement promoted heart failure and ischemic injury by impairing glucose metabolism and induce mitochondrial oxidative stress [18,33]. Therefore, further evidence is needed to elucidate the physiological and pathological functions of BCAAs in different tissues and conditions.

There are two aminotransferases for BCAAs; namely, BCAT1 in the cytosol and BCAT2 in the mitochondria [12]. We observed that *Bcat1* but not *Bcat2* was down-regulated in the brain tissues from diabetic, aged, or AD mice. Down-regulation of *Bcat1* would result in the accumulation of BCAAs in cells the tissues, which is a core mechanism underlying several types of cancer, including leukemia [34], nonsmall cell lung cancer [13], pancreatic adenocarcinoma [35], and breast cancer [36]. For instance, BCAT1 exerts its oncogenic function through BCAA production of leukemia by activating mTOR signaling [35]. Indeed, we observed that BCAA supplement in the diet activated mTOR signaling in the brain tissues, which may be due to the effects of leucine, an activator for mTOR signaling. Importantly, we showed that BCAA supplement promoted the phosphorylation of Tau protein in the brains of 3xTg AD mice, which was further confirmed in isolated neurons treated with leucine or *Bcat1* knockdown. Our results showed that these effects of leucine and *Bcat1* were largely dependent upon the activation of mTOR, which was previously reported to phosphorylate Tau protein directly [21]. However, BCAAs and *Bcat1* did not change the content of β-amyloid peptides in the brain tissues of AD mice.

In conclusion, our findings implicate that BCAAs may be one of the mechanisms connecting diabetes and AD. BCAA serum levels are increased in patients with diabetes and AD and supplement of BCAA in the diet promoted the development of AD by regulating the accumulation of phosphorylated Tau protein in an mTOR-dependent manner.

## Supporting information

**Supplementary Figure 1:** The normalized level of lysine in type 2 diabetes (T2D, A), Alzheimer’s disease (AD, B), and aged donors C). n=6-10 in each group. The cases are the same from that of Figure 1.

## Abbreviations

AD: Alzheimer’s disease
BCAA: branched-chain amino acid
BCAT1: branched-chain amino acid transaminase 1
IDE: insulin degrading enzyme
mTOR: mammalian target of rapamycin
MWM: Morris water maze
PR: protein-restricted
NOR: novel object recognition
