# Supplementary material for: Defect of branched-chain amino acid metabolism promotes the development of Alzheimer’s disease by targeting the mTOR signaling
Source: Biosci Rep. 2018 Jul 3;38(4):BSR20180127. doi: 10.1042/BSR20180127 (PMC6028749; doi:10.1042/BSR20180127)
Supplement: Supplementary file 1 [file bsr20180127_Supp1.pdf]

Supplementary data

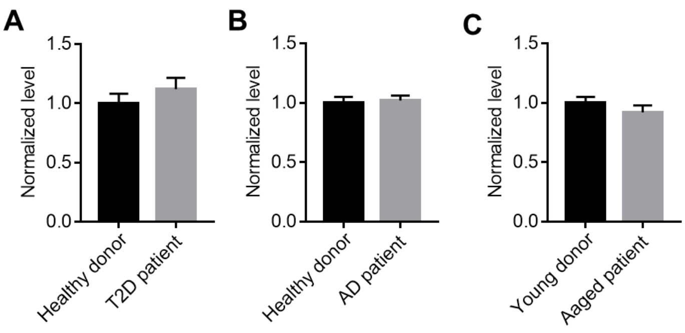

Supplementary Figure 1 The normalized level of lysine in type 2 diabetes (T2D, A), Alzheimer's disease (AD, B), and aged donors (C). n=6-10 in each group. The cases are the same from that of Figure 1.
